# Warming increases the differences among spring phenology models under future climate change

**DOI:** 10.3389/fpls.2023.1266801

**Published:** 2023-10-23

**Authors:** Yunhua Mo, Xiran Li, Yahui Guo, Yongshuo Fu

**Affiliations:** ^1^ College of Water Sciences, Beijing Normal University, Beijing, China; ^2^ College of Urban and Environmental Sciences, Central China Normal University, Wuhan, China

**Keywords:** spring phenological model, PEP725, SSP scenarios, climate change, future prediction

## Abstract

Phenological models are built upon an understanding of the influence of environmental factors on plant phenology, and serve as effective tools for predicting plant phenological changes. However, the differences in phenological model predictive performance under different climate change scenarios have been rarely studied. In this study, we parameterized thirteen spring phenology models, including six one-phase models and seven two-phase models, by combining phenological observations and meteorological data. Using climatic data from two Shared Socioeconomic Pathways (SSP) scenarios, namely SSP126 (high mitigation and low emission) and SSP585 (no mitigation and high emission), we predicted spring phenology in Germany from 2021 to 2100, and compared the impacts of dormancy phases and driving factors on model predictive performance. The results showed that the average correlation coefficient between the predicted start of growing season (SOS) by the 13 models and the observed values exceeded 0.72, with the highest reaching 0.80. All models outperformed the NULL model (Mean of SOS), and the M1 model (driven by photoperiod and forcing temperature) performed the best for all the tree species. In the SSP126 scenario, the average SOS advanced initially and then gradually shifted towards a delay starting around 2070. In the SSP585 scenario, the average SOS advanced gradually at a rate of approximately 0.14 days per year. Moreover, the standard deviation of the simulated SOS by the 13 spring phenology models exhibited a significant increase at a rate of 0.04 days per year. On average, two-phase models exhibited larger standard deviations than one-phase models after approximately 2050. Models driven solely by temperature showed larger standard deviations after 2060 compared to models driven by both temperature and photoperiod. Our findings suggest investigating the release mechanisms of endodormancy phase and incorporating new insights into future phenological models to better simulate the changes in plant phenology.

## 1 Introduction

Global climate is undergoing unequivocal unprecedented changes, including variations in extreme events that can be attributed to the influence of human activities (Masson-Delmotte et al., 2021). Since the 1970s, there has been a clear warming trend. The Earth’s temperature is currently experiencing rapid increasing, with the period 2016 to 2020 being the warmest five years since 1850 (Masson-Delmotte et al., 2021). One of the consequences of global warming is the alteration of vegetation phenology (Brown et al., 2012; Oberbauer et al., 2013; Park et al., 2015; Mo et al., 2019). Plant phenology studies the cyclic variations in plant activities and their relationship with environmental conditions (Cleland et al., 2007). Phenological events such as leaf unfolding, flowering, fruiting, and leaf senescence are external manifestations of plant physiological activities, regulated by seasonal changes in temperature, precipitation, and other environmental factors (Shen et al., 2022). As a significant characteristic of climate change, warming often leads to an earlier onset of spring phenology in plants (Piao et al., 2006). Investigating changes in plant phenology can enhance understanding of the impacts of climate change, thereby providing insights for climate change mitigation.

As a sensitive bioindicator of climate change, variations in vegetation phenology have extensive effects on the structure and functioning of terrestrial ecosystems (Richardson et al., 2013). At the individual scale, proper timing of dormancy allows plants to avoid frost damage, which is crucial for their survival and growth (Gu et al., 2008). At the population scale, an increase in the asynchrony of phenology would reduce inter-species competition for resources within plant communities and promote species coexistence (Menzel, 2002). At the community scale, phenology influences the ecological niches of interacting species (Chuine, 2010). At the ecosystem scale, vegetation phenology regulates material cycles (such as carbon and water) and energy fluxes within the ecosystem (Richardson et al., 2012). Additionally, plant phenology influences surface albedo and canopy conductance, exerting feedback on the climate system (Richardson et al., 2013). Therefore, investigating changes in plant phenology under climate-warming conditions is of great significance.

Plant dormancy is one of the key phenological stages. It can be categorized into three phases: paradormancy, endodormancy, and ecodormancy (Lang et al., 1987). In the paradormancy phase, growth inhibition is primarily caused by physiological factors outside the plant bud. In the endodormancy phase, the quiescent state is mainly induced by the dormancy structures themselves, and plants in this stage require the chilling accumulation. In the ecodormancy phase, the quiescent state is primarily influenced by external environmental factors, and plants in this stage require the accumulation of heat units (forcing) (Lang et al., 1987; Kramer, 1994; Basler, 2016). Based on these mechanisms, several environmental factor-driven phenological models have been developed for predicting phenological changes. One of the earliest spring phenology model can be traced back to 1735, driven solely by temperature and involving the accumulation of heat units during the ecodormancy phase, referred to as the Thermal Time model (Reaumur, 1735). Considering the influence of photoperiod on the ecodormancy phase, the photothermal time model was developed (Masle et al., 1989). Besides, these phenological models can also be divided into two categories based on the number of dormant phases they simulate. Models that exclusively account for the release during the ecodormancy phase are referred to as one-phase models, while models that account for both the release during the endodormancy and ecodormancy phases are referred to as two-phase models. In two-phase models, the sequential model refers to forcing accumulation occurring after chilling accumulation, the parallel model involves concurrent accumulation of chilling and forcing (Hänninen, 1990; Kramer, 1994), and the alternating model determines whether chilling or forcing accumulation occurs based on the daily mean temperature (Murray et al., 1989). The temperature response of the endodormancy phase can be a triangular or bell-shaped function, while the temperature response of the ecodormancy phase can be a linear or sigmoid function (Basler, 2016). The differences in environmental driving factors and dormant phases within phenological models may have an impact on their simulation performance, particularly in the context of climate warming.

The previous study indicates that under current climate conditions, both one-phase and two-phase models exhibit similar simulation performance due to the fulfillment of chilling requirements (Vitasse et al., 2011). The differences in temperature response functions, particularly the chilling response function, have limited impacts on the simulation performance of the models (Mo et al., 2023). However, with continued climate warming, meeting chilling requirements may confront more challenges in the future. Therefore, it is crucial to compare the performance of one-phase and two-phase models under climate change, especially warming scenarios. In a climate warming scenario, it is expected that spring phenology will advance. However, photoperiod reduces the sensitivity of plants to warming, preventing the onset of spring phenology from occurring too early and avoiding the impacts of frost (Fu et al., 2019). Additionally, photoperiod advances spring phenology, maximizing resource acquisition for plants (Meng et al., 2021). There exist complex interactions between temperature and photoperiod, and it remains largely unknown whether existing models can capture these mechanisms. Therefore, a thorough comparison among phenological model performances under various scenarios can be very informative.

In this study, the parameterization of 13 spring phenology models was conducted by combining phenological observation data with meteorological data. The spring phenology models consisted of six one-phase models and seven two-phase models. Subsequently, using data from two Shared Socioeconomic Pathways (SSPs) scenarios, namely, SSP126 and SSP585, future spring phenology from 2021 to 2100 was predicted, and the impacts of driving factors and dormancy phases on the predictive performance of the models were compared. The objectives of this research were: (1) to assess the changes in spring phenology under different future scenarios; (2) to compare the influences of driving factors and dormancy phases on the predictive performance of the models under climate change scenarios.

## 2 Materials and methods

### 2.1 Study region and Phenological observations

**Figure 1:**
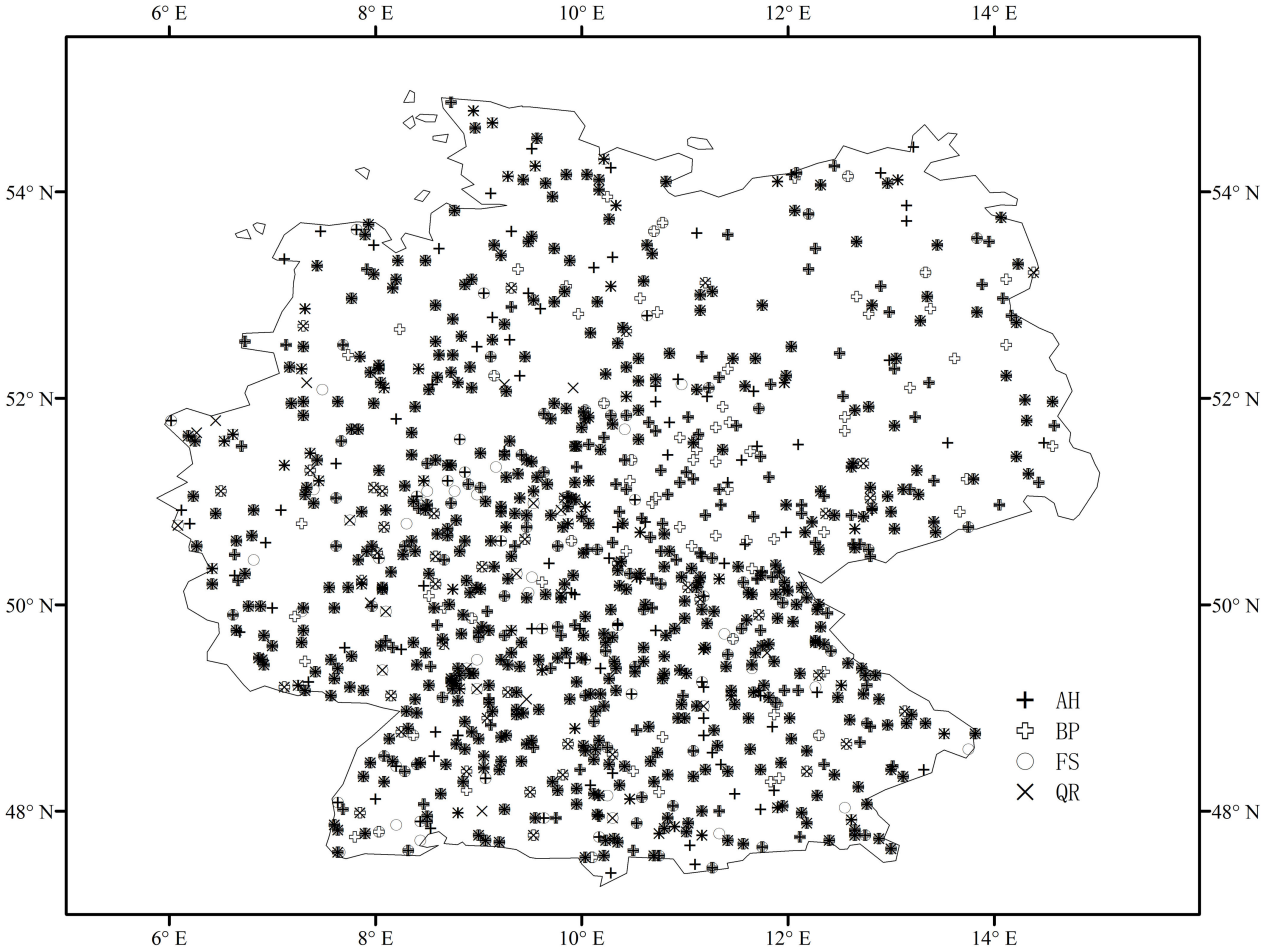
Spatial distribution of phenological sites for four tree species in Germany: Aesculus hippocastanum (AH), Betula pendula (BP), Fagus sylvatica (FS), and Quercus robur (QR).

Our study region is located in Germany, with typically temperate to alpine climate. The corresponding phenological observations were collected from the Pan European Phenology (PEP) project. The PEP project maintains and develops the Pan European Phenological Database (PEP725), which has open access to science and education, aimed at promoting phenological research, education, and environmental monitoring (Templ et al., 2018). PEP725 records phenological dates in day-of-the-year (Julian day) and phenological codes in Biologische Bundesanstalt, Bundessortenamt und Chemische Industrie (BBCH) scale (Meier, 2001). We selected records with BBCH codes 10 and 11 as spring phenology (the start of growing season, SOS hereafter). To obtain reliable spring phenology, we selected phenological records according to the following criteria: (1) both leaf unfolding and leaf senescence are available in the same year, and the leaf senescence date is greater than the leaf unfolding date, (2) sites have been available for observation for no less than 40 years, and (3) the number of sites for each tree species is greater than 100. As a result, we used records of four tree species, they are Aesculus hippocastanum (AH), Betula pendula (BP), Fagus sylvatica (FS), and Quercus robur (QR). The number of phenological sites for AH, BP, FS, and QR is 814, 770, 586, and 592, respectively. The spatial distributions of the selected phenological sites for the four tree species are shown in **Figure 1**.

### 2.2 Meteorological data

#### 2.2.1 E-OBS gridded dataset

The meteorological data for the model parameterization in this study were obtained from the E-OBS dataset. It is a high-resolution gridded dataset of daily climate over Europe, developed by the EU-funded ENSEMBLES project (Hofstra et al., 2009). E-OBS dataset is constructed by interpolating the wider range of Europe’s most complete station data (Klok and Klein Tank, 2009) and is intended to support the validation of Regional Climate Models (RCMs) and climate change research (Schrier et al., 2013). The dataset contains two spatial resolutions of 0.1 and 0.25°, covering 25° N~71.5°N; 25°W ~ 45°E. It provides daily mean temperature (TG), daily minimum temperature (TN), daily maximum temperature (TX), daily precipitation sum (RR), etc., since 1950 January 1st to present. Here, we used the daily mean temperature data for E-OBS v23.1e at 0.1°, covering the period from 1950-01-01 to 2020-12-31.

#### 2.2.2 Climate change scenarios dataset

The climate change scenarios used in this study are derived from the Scenario Model Intercomparison Project (ScenarioMIP) of Phase 6 of the Coupled Model Intercomparison Project (CMIP6) (O’Neill et al., 2016). ScenarioMIP provides a marix of multiple shared socioeconomic pathways (SSPs) and radiative forcing levels (Van Vuuren et al., 2012; O’Neill et al., 2016). SSPs describe different socioeconomic change pathways resulting from different development strategies and are used to estimate greenhouse gas emissions. They include five pathways: SSP1 (Sustainability), SSP2 (Middle of the road), SSP3 (Regional rivalry), SSP4 (Inequality), and SSP5 (Fossil-fuel development) (Meinshausen et al., 2020). The radiative forcing levels by 2100 range from low to high, specifically 2.6 W/m², 4.5 W/m², 7.0 W/m², and 8.5 W/m². In this study, we employed two climate change scenarios, namely SSP1-2.6 (SSP126) and SSP5-8.5 (SSP585). The SSP126 simulated the scenario with high mitigation and low emission. The SSP585 simulated no mitigation and high emission. Therefore, our simulations could illustrate the differences of SOS under the sustainable pathway and the energy-intensive pathway. To reduce uncertainty, we computed the ensemble mean of 13 CMIP6 climate models. Information on these climate models is summarized in **Supplementary Table 1**.

### 2.3 Spring phenological model

This study utilized 13 spring phenology models to analyze the performance of phenology models under different climate change scenarios. Among these models, there are six one-phase models and seven two-phase models, with five models driven solely by temperature and eight models driven by both temperature and photoperiod (**Table 1**). The chilling temperature response functions include triangular and bell-shaped functions, while the forcing temperature response functions include linear and sigmoid functions. The formulae employed by the 13 models and the meanings of the parameters within the formulae are summarized in **Supplementary Tables 2, 3**, respectively.

**Table 1:** Summary of the spring phenology models.

Model abbreviation^1^	Full model name	release	Drivers^2^	Comments/References
**NULL**	NULL model			Mean of the SOS^3^
**TT**	Thermal Time model	Ecodormancy release	F	(Kramer, 1994; Chuine et al., 1999; Reaumur, 1735)
**TTs**	Thermal Time model	Ecodormancy release	F	(Hänninen, 1990; Kramer, 1994)
**PTT**	Photothermal Time model	Ecodormancy release	PF	(Masle et al., 1989)
**PTTs**	Photothermal Time model	Ecodormancy release	PF	(Landsberg, 1974; Črepinšek et al., 2006; Basler, 2016)
**M1**	M1 model	Ecodormancy release	PF	(Blümel and Chmielewski, 2012)
**M1s**	M1 model	Ecodormancy release	PF	M1 model using a sigmoid temperature response for forcing
**AT**	Alternating model	Endo- and ecodormancy releases	CF	(Murray et al., 1989)
**SM1**	Sequential model (M1 variant)	Endo- and ecodormancy releases	CPF	(Basler, 2016)
**SM1b**	Sequential model (M1 variant)	Endo- and ecodormancy releases	CPF	SM1 model using a bell-shaped temperature response for chilling
**PA**	Parallel model	Endo- and ecodormancy releases	CF	(Hänninen, 1990; Kramer, 1994)
**Pab**	Parallel model	Endo- and ecodormancy releases	CF	PA model using a bell-shaped temperature response for chilling
**PM1**	Parallel M1 model	Endo- and ecodormancy releases	CFP	(Basler, 2016)
**PM1b**	Parallel M1 model	Endo- and ecodormancy releases	CFP	PM1 model using a bell-shaped temperature response for chilling

^1^In the model abbreviations: s: using a sigmoid temperature response function for forcing, otherwise employing a growing-degree-day temperature response function; b: using a bell-shaped temperature response function for chilling, otherwise employing a triangular temperature response function.

^2^Driver abbreviations: C: chilling temperature, F: forcing temperature, and P: photoperiod.

^3^SOS: start of the growing season.

### 2.4 Calculation of the photoperiod

The magnitude of the photoperiod varies with spatial location and date. This study adopts the approach outlined in http://herbert.gandraxa.com/length_of_day.xml, utilizing latitude and day of the year to calculate the photoperiod. The calculation formula is as follows:


(1) $$\begin{array}{l} axis=\frac{\pi }{180}*23.439\\ m=1-\text{tan}(lat)*\text{tan}(axis*\text{cos}(\frac{\pi *doy}{182.625}))\\ b=\frac{\arccos(1-m)}{\pi }\\ photoperiod=24*b \end{array}$$

where ‘axis’ represents the obliquity of the ecliptic, arising from the Earth’s rotation axis not being perpendicular to its orbital plane. This phenomenon results in the equatorial plane deviating from parallel alignment with the ecliptic plane, forming a constant angle of 23.439° in our study, with gradual changes occurring only over millennia. ‘m’ signifies the exposed radius part between sun’s zenith and sun’s circle. ‘b’ represents the fraction of the Sun’s circular exposed, a parameter dependent on factors including geographical latitude (‘lat’) and the day of the year (‘doy’). ‘doy’ denotes the day’s position within the year, taking values between 1 and 365 (or 366 in leap years).

### 2.5 Model calibration and prediction

We used 80% of the observed SOS from the middle of the available years to build the spring phenological models, reserving the remaining 20% of the SOS – which were from the beginning and the end of the available years - for evaluating the predictive performance of the models. For instance, if phenological observation data were available for the years 1961-2000 at a given site, we chose data from 1965-1996 for model parameterization and used data from 1961-1964 as well as 1997-2000 for model validation. Considering the model’s application in forecasting, we adopted this data partitioning approach, which is more conducive to extrapolation beyond the years covered. Considering that all sites have a minimum of 40 years of data, each site has a minimum of 32 years of modeling data and at least 8 years of validation data. We employed generalized simulated annealing (GenSA) for model parameter optimization (Xiang et al., 2013), and this work was based on the PHENOR modeling framework (Hufkens et al., 2018).

Root Mean Square Error (RMSE) and Akaike Information Criterion (AIC) (Akaike, 1974) were used to assess the goodness of fit and to select the best models. RMSE is commonly used to measure prediction deviation and reflects the concentration of data around the best-fit line, with smaller values indicating higher model accuracy. RMSE can be calculated using the following formula:


(2) $$RMSE=\sqrt{\frac{\sum_{i=1}^{n}{\left(observed_{i}-predicted_{i}\right)}^{2}}{n}}$$

where *observed*_i_ and *predicted*_i_ are the *i*-th observation and the model prediction, respectively, and *n* is the number of observations.

AIC can identify the best model that provides a good explanation of the data with the fewest parameters, as well as preventing overfitting. AIC can be calculated using the following formula:


(3) $$AIC=n*\log\left(RMSE^{2}\right)+2k+\frac{2k(k+1)}{n-k-1}$$

where *k* is the number of free parameters to be fitted in the model.

We employed E-OBS meteorological data and phenological observations for model parameterization and validation. After selecting the optimal parameters, we utilized climate change scenario data as model inputs to predict and analyze model differences under different scenarios.

## 3 Results

Overall, the spring phenology simulated by the 13 models (**Supplementary Figures 1A-M**) has a similar spatial pattern to the PEP725 observational records (**Supplementary Figure 1N**). Among the four tree species, AH and BP had the earliest growing season start dates, with an average SOS of about 110 days, while QR had the latest growing season start date, about 123 ± 7 days.

**Figure 2:**
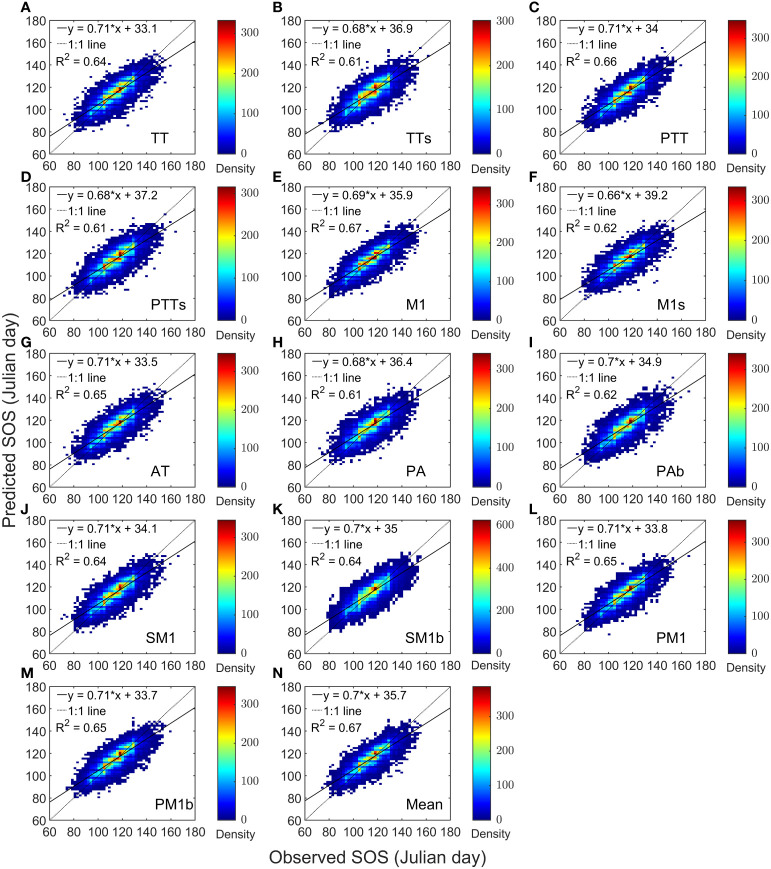
Heat maps of model predictions and PEP725 phenological observations for four tree species. Panels **(A–M)** are compartisons of each model separately. For Panel **(N)**, the predicted start of growing season (SOS) is the mean of the 13 model predictions.

We performed a linear regression between the predictions of 13 models and the observed values separately. The coefficient of determination (R^2^) of the regression models ranged from 0.61 to 0.67 (**Figures 2A-M**), with the R^2^ of the model-mean as 0.67 (**Figure 2N**). The slopes varied from 0.66 to 0.71, with an average of 0.7. Overall, the average correlation coefficient between the predicted and observed values of the average SOS across the 13 models exceeded 0.7, reaching a maximum of 0.8 (**Supplementary Figure 2**). For AH, the correlation coefficients between the predicted and observed SOS ranged from 0.74 to 0.78; for BP, the range was 0.76 to 0.8; for FS, it was 0.63 to 0.72; and for QR, it was 0.71 to 0.77.

Regarding the four tree species, the performances of the 13 models were superior to the NULL model (**Table 2**). Among these models, the M1 model performed the best for all of the tree species, achieving the lowest RMSE and AIC values. However, for AH, FS, and QR, the TTs model exhibited the poorest performance, as it obtained the highest RMSE and AIC values. For BP, the SM1 model performed the worst.

**Table 2:** Summary of the comparison between the simulated start of growing season (SOS) and PEP725 phenological observation of 13 models for the four tree species: Aesculus hippocastanum (AH), Betula pendula (BP), Fagus sylvatica (FS), and Quercus robur (QR).

models	AH		BP		FS		QR	
	RMSE(days)	AIC	RMSE(days)	AIC	RMSE(days)	AIC	RMSE(days)	AIC
NULL	12.1		11.3		9.6		11.6	
TT	7.9	30787	7.3	27862	7.1	21003	7.7	21906
TTs	8.3	31516	7.5	28286	7.6	21712	8.3	22610
PTT	7.8	30561	7.0	27356	7.0	20879	7.5	21626
PTTs	8.3	31466	7.4	28129	7.5	21650	8.1	22382
M1	7.7	30360	6.9	26978	6.8	20510	7.5	21602
M1s	8.2	31231	7.3	27912	7.3	21330	8.0	22214
AT	7.9	30725	7.2	27737	7.1	21036	7.6	21778
SM1	8.3	31479	7.6	28397	7.4	21525	8.1	22384
SM1b	8.3	31380	7.5	28299	7.4	21522	8.1	22371
PA	8.0	30855	7.2	27744	7.0	20943	7.8	21989
PAb	7.9	30764	7.3	27793	7.1	21063	8.0	22221
PM1	7.8	30577	7.1	27528	7.0	20959	7.7	21796
PM1b	7.9	30647	7.1	27455	7.1	21002	7.6	21717

The models are represented by abbreviations, as listed in **Table 1**, and their performance was evaluated using root mean squared error (RMSE), and Akaike information criterion (AIC).

**Figure 3:**
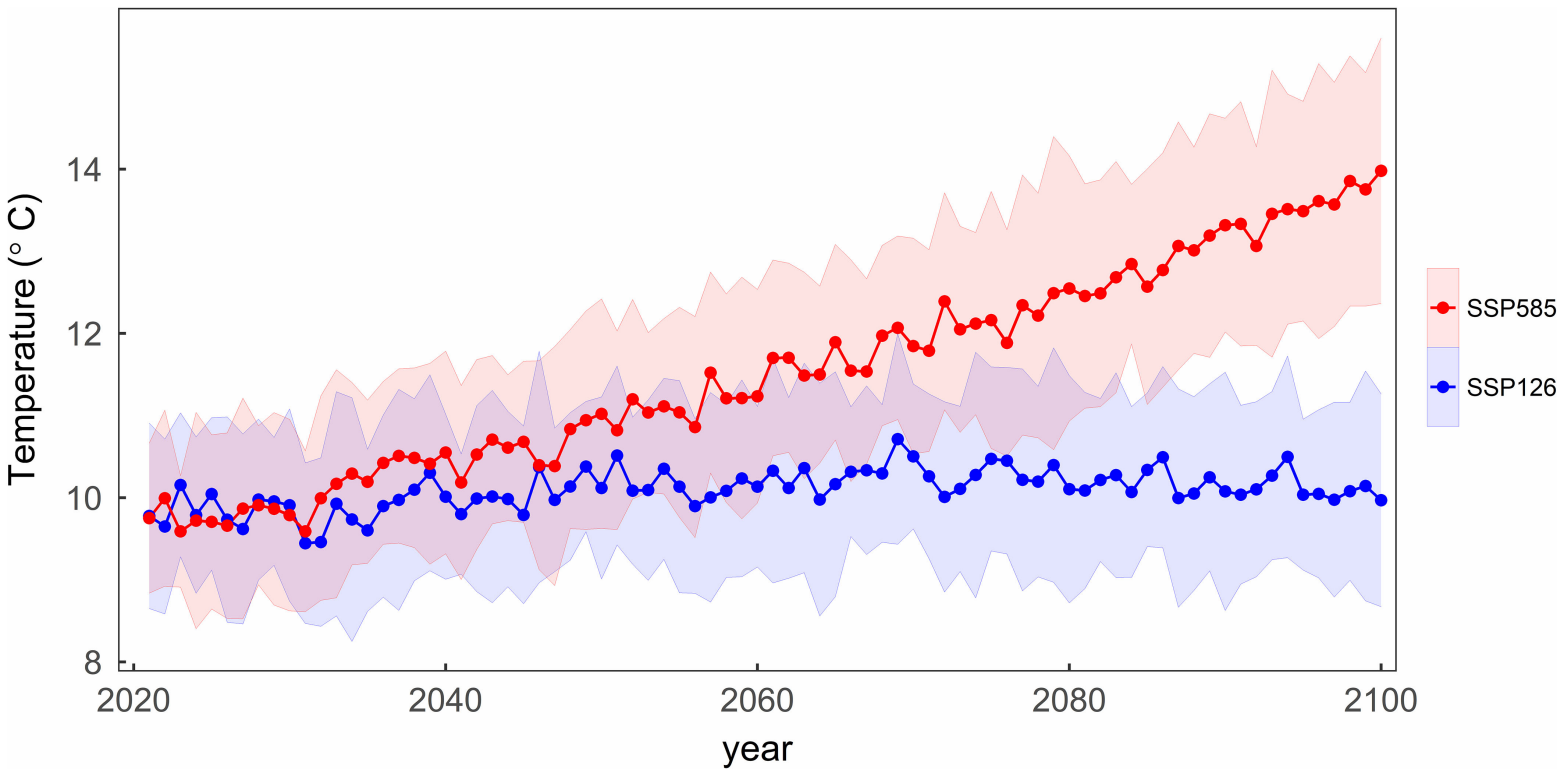
The variation of the mean temperature with the years under two scenarios in the study area. The shading represents one standard deviation of temperature from thirteen CMIP6 climate models under each scenario.

Since most of the phenological observation sites for the four tree species are coincident, the mean temperatures in their regions have similar changes over the years. **Figure 3** illustrates the average temperature changes across all sites for four tree species during the period from 2021 to 2100 under two SSP scenarios. Under the SSP126 scenario, the average temperature initially rises and then gradually decreases, with a turning point around 2070. In contrast, under the SSP585 scenario, the average temperature gradually increases, results in the study area over 4°C warmer than under the SSP126 in 2100.

**Figure 4:**
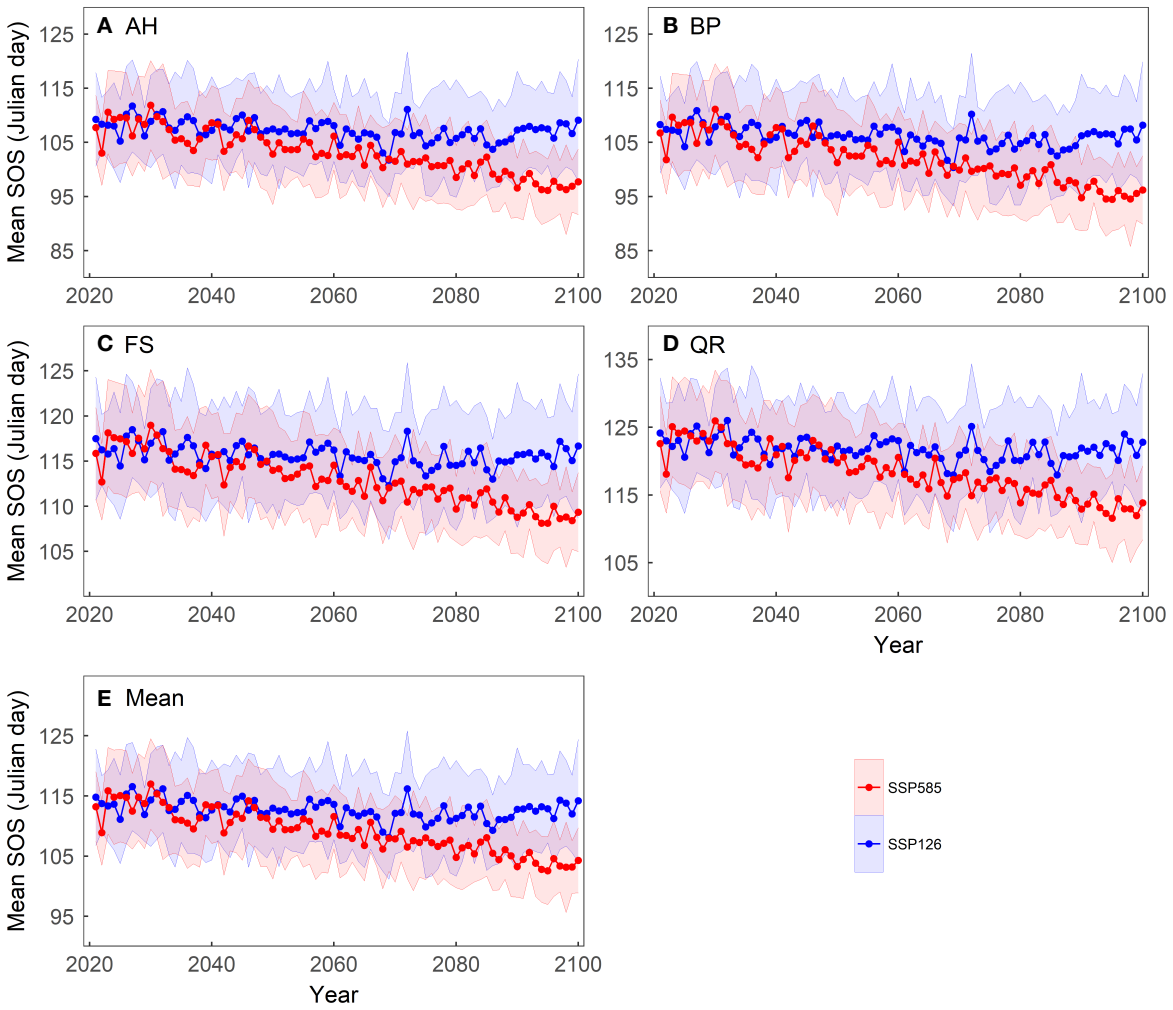
Changes in the mean start of growing season (SOS) of four tree species over the period 2021-2100 under two scenarios: **(A)** Aesculus hippocastanum (AH), **(B)** Betula pendula (BP), **(C)** Fagus sylvatica (FS), **(D)** Quercus robur (QR) and **(E)** their mean. The shading represents one standard deviation of the predicted SOS based on temperature data from thirteen CMIP6 climate models for each scenario.

We computed the average of the simulated results from the 13 spring phenology models to reflect the phenological changes under the two scenarios (**Figure 4**). Under the SSP126 scenario, the average SOS first advances and then gradually delays, with a turning point around 2070. In contrast, under the SSP585 scenario, the average SOS progressively advances, at a rate of approximately 0.14 days per year. Although the spring phenology of the four tree species differs, they exhibit similar trends under both scenarios. Under the SSP585 scenario, among the four tree species, BP exhibits the largest advance in SOS, approximately 0.16 days per year, while FS shows the smallest advance, approximately 0.11 days per year.

**Figure 5:**
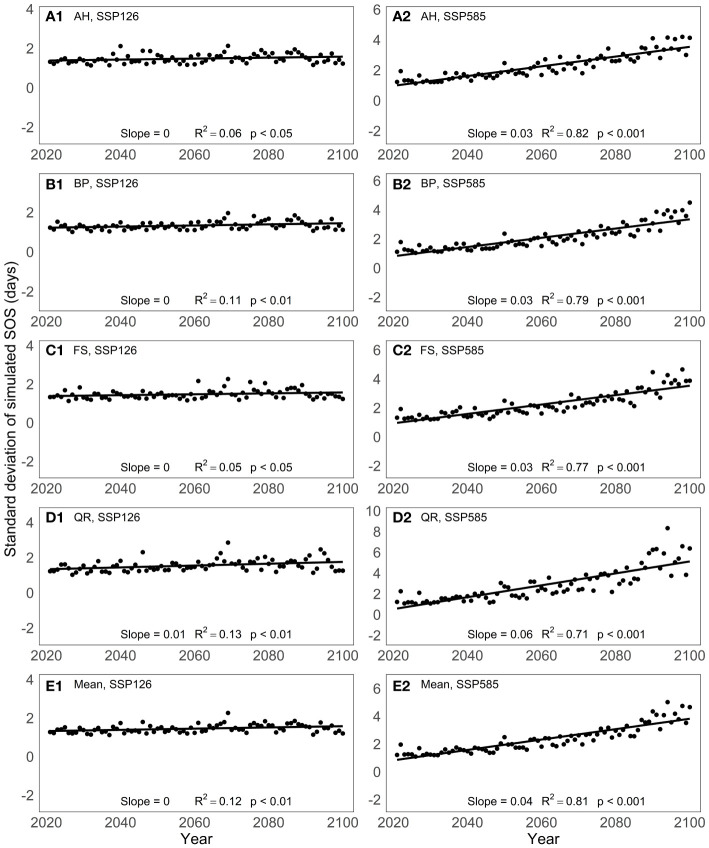
Changes in the standard deviation of the predictive results of 13 spring phenology models under two scenarios between 2021 and 2100: **(A)** Aesculus hippocastanum (AH), **(B)** Betula pendula (BP), **(C)** Fagus sylvatica (FS), **(D)** Quercus robur (QR) and **(E)** their mean.

To analyze the predictive differences among models, we computed the standard deviation of the predicted SOS from the 13 spring phenology models for the period from 2021 to 2100 (**Figure 5**). Under the SSP126 scenario, the standard deviation of the model-predicted SOS remains relatively constant over the years for the four tree species studied. However, under the SSP585 scenario, the standard deviation of the simulated SOS by the 13 spring phenology models exhibits a significant increase from 2021 to 2100, with an average increase of 0.04 days per year. Among the four tree species, the SOS standard deviation shows the greatest variation for QR, with a magnitude of 0.06 days per year, while the other three tree species exhibit a variation of 0.03 days per year.

**Figure 6:**
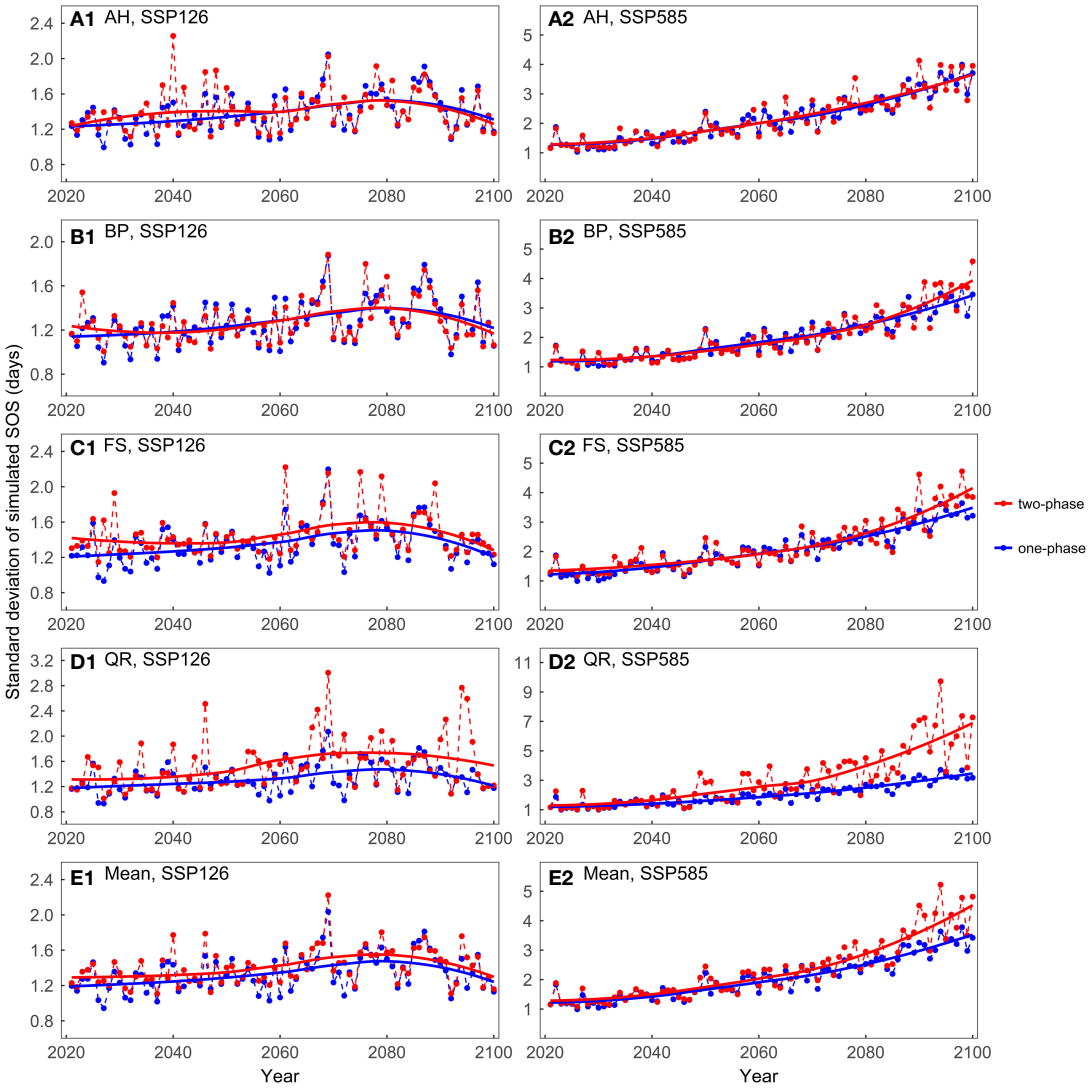
Comparing the standard deviations of the predicted start of growing season (SOS) for the four tree species considering different dormancy release scenarios: **(A)** Aesculus hippocastanum (AH), **(B)** Betula pendula (BP), **(C)** Fagus sylvatica (FS), **(D)** Quercus robur (QR), and **(E)** their mean. One-phase represents the one-phase models, which solely explains ecodormancy release, while two-phase represents two-phase models, which explains both endodormancy and ecodormancy release.

Overall, across both scenarios, the mean values of the predicted SOS were very similar between the one-phase and two-phase models (**Supplementary Figure 3**). In the SSP126 scenario, the standard deviations of the model predictions were generally smaller than 2 days, with slightly larger standard deviations observed for the two-phase models (**Figure 6**). In the SSP585 scenario, on average, the two-phase models exhibited larger standard deviations after approximately 2050. For BP, FS, and QR, as temperatures increased, the two-phase models exhibited greater prediction differences than one-phase models.

**Figure 7:**
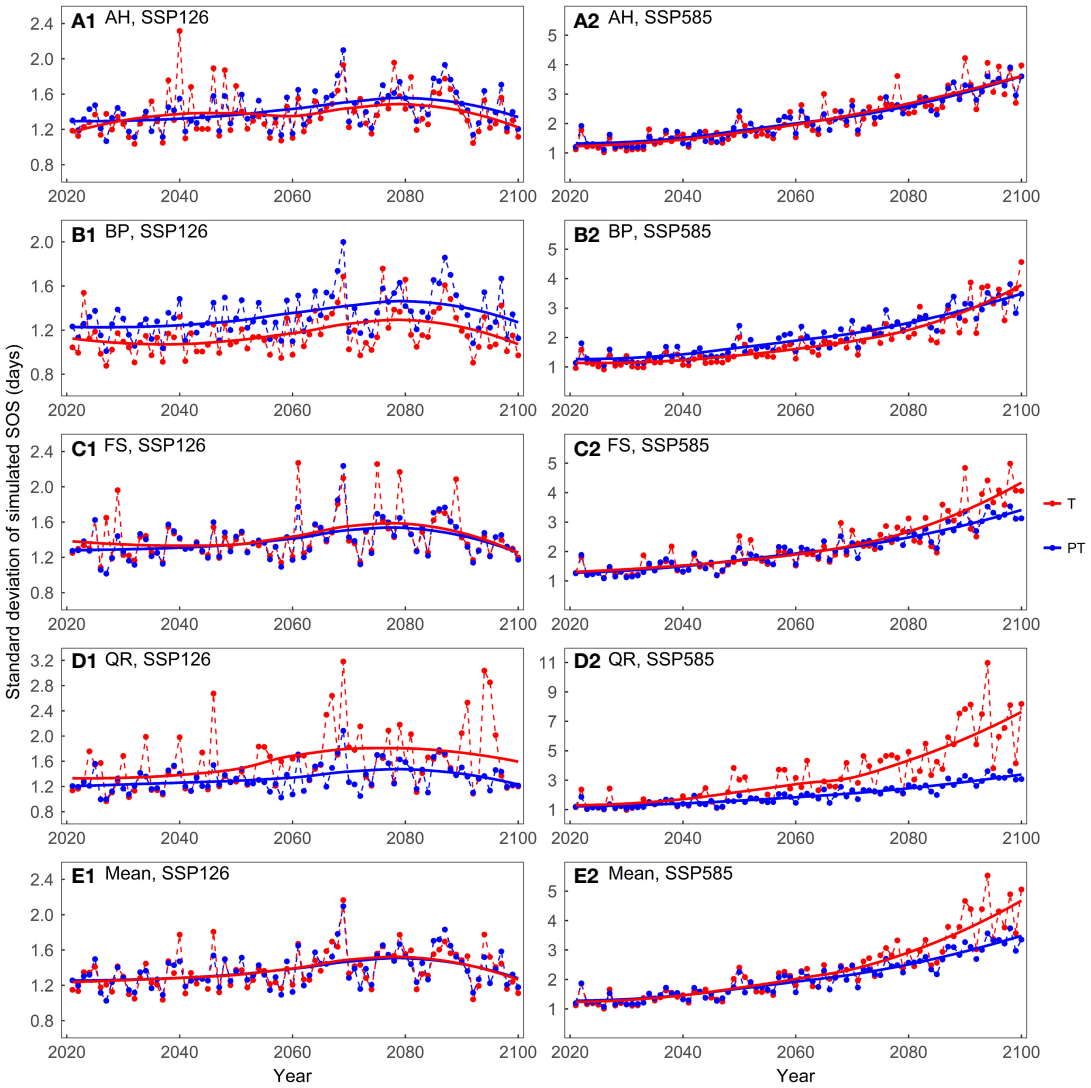
Comparing the standard deviations of the predicted start of growing season (SOS) for the four tree species driven by different factors: **(A)** Aesculus hippocastanum (AH), **(B)** Betula pendula (BP), **(C)** Fagus sylvatica (FS), **(D)** Quercus robur (QR), and **(E)** their mean. T represents models driven solely by temperature, while PT represents models driven by both temperature and photoperiod simultaneously.

The mean of the predicted SOS was similar between the temperature-driven models and the temperature and photoperiod-driven models, across both scenarios (**Supplementary Figure 4**). In the SSP126 scenario, the standard deviations of the model predictions were generally small and exhibited a trend of initially increasing and then decreasing (**Figure 7**). In the SSP585 scenario, on average, the temperature-driven models showed larger standard deviations after 2060. For FS and QR, as temperatures increased, the temperature-driven models showed greater prediction differences in the SSP585 scenario.

## 4 Discussion

We parameterized and validated 13 spring phenology models using E-OBS meteorological data and phenological observations. The results indicate that model simulations exhibit spatial patterns similar to phenological observations, with correlation coefficients exceeding 0.72 and reaching as high as 0.8. The RMSE values for all 13 models are lower than those of the NULL model, with the M1 model being identified as the best predictive model for spring phenology among the four tree species, in agreement with Basler’s study (Basler, 2016).

To investigate the sources of model prediction discrepancy, we quantitatively examined the differences due to model structures (i.e. one-phase vs two-phase models) and driving factors (i.e. only temperature vs temperature and photoperiod).

Firstly, we compared the influence of dormancy release on the predictive performance of the models. Our research demonstrates that one-phase models and two-phase models exhibit similar performance. Previous studies have also reached similar conclusions (Hänninen and Kramer, 2007; Linkosalo et al., 2008; Vitasse et al., 2011; Basler, 2016), including our previous research (Mo et al., 2023). Both the one-phase and two-phase models involve the process of heat accumulation, with the main difference being that the two-phase model additionally incorporates the process of chilling accumulation. Temperature manipulation experiments indicate that chilling temperature can affect the spring phenology of certain tree species (Murray et al., 1989; Heide, 1993a; Heide, 1993b). Therefore, when there are issues in meeting the chilling requirement, the two-phase model can demonstrate better performance. Our results imply that the chilling requirement of the four tree species in our study area can be met under current climate conditions, which is consistent with previous research findings (Vitasse et al., 2011; Basler, 2016). However, under future climate warming scenarios, the fulfillment of chilling requirements may face challenges.

The two-phase model used in this study consists of sequential, parallel, and alternating models. The sequential model posits that the chilling requirement must be fulfilled before heat accumulation begins. The parallel model suggests that chilling accumulation and heat accumulation can occur simultaneously, while the alternating model proposes that the choice between chilling accumulation and heat accumulation depends on temperature conditions. Under the SSP585 scenario, the standard deviation of the two-phase model is generally higher than that of the one-phase model, particularly after 2050. This may be attributed to the increasing temperature, which poses challenges in meeting the chilling requirement and highlights the importance of chilling accumulation in the two-phase model, whereas the one-phase model assumes that the chilling requirement has already been satisfied. Furthermore, the differences among the two-phase models can be further amplified by variations in the chilling temperature response function employed in each model.

We then compared models driven solely by temperature and those driven by both temperature and photoperiod. When considering about the mechanisms of how climate factors influence spring phenology, for temperate and boreal regions, the primary factor is the temperature, as well as the photoperiod (Flynn and Wolkovich, 2018; Fu et al., 2019). The temperature has a dual impact on spring phenology (Chuine et al., 2016). On one hand, plants require chilling accumulation during winter to release the endodormancy phase, and on the other hand, they need to accumulate heat (experiencing forcing temperatures) to release the ecodormancy phase. Studies have indicated that the fulfillment of chilling requirements affects the demand for heat accumulation (Murray et al., 1989; Carter et al., 2017). In addition to temperature, photoperiod plays a role in regulating spring phenology. When warming advances the SOS, photoperiod then delays it to avoid plant exposure to frost. When temperature variation delays the SOS, photoperiod triggers an advance to provide more time for photosynthesis (Meng et al., 2021).

These response characters have been captured in our simulations. Our results indicate that, under the SSP585 scenario, models that simultaneously consider the effects of both temperature and photoperiod have smaller prediction differences in forecasting the spring phenology of FS and QR, compared to models that only consider temperature effects. However, there were no significant differences for AH and BP. This may be attributed to the varying sensitivity of different tree species’ spring phenology to photoperiod. It has been shown that a 4°C increase in temperature can limit the advancement of SOS in FS due to photoperiod restrictions, while SOS in AH is not limited by photoperiod (Fu et al., 2019). This characteristic of photoperiod contributes to smaller prediction differences in models that consider photoperiod effects, which aligns with the findings of this study. With ongoing climate warming, the spring phenology of photoperiod-sensitive tree species may experience greater impacts.

Comprehensively, our results show that the average predicted SOS of the two-phase model and the one-phase model are relatively close under both scenarios (**Supplementary Figure 3**), suggesting the need for further investigation of the endodormancy phase release mechanism. Additionally, it has been shown that the lack of endodormancy break date data often leads to inaccurate predictions of endodormancy break dates and can result in substantial differences in prediction outcomes under warming scenarios (Chuine et al., 2016). When warming reaches a certain level, photoperiod restricts the advancement of plant spring phenology, leading to predictions from models that consider both temperature and photoperiod to be higher than those from models that only consider temperature. However, our results do not support this expectation (**Supplementary Figure 4**). Therefore, there is still uncertainty regarding the synergistic effects of temperature and photoperiod on plant spring phenology, and further research, such as manipulation experiments, is needed to improve existing spring phenology models.

Furthermore, some previous studies have suggested that temperature exhibits asymmetric effects on spring phenology, including our previous research (Mo et al., 2023). In this study, we also considered the asymmetric impact of temperature and parameterized the model using daily mean temperature, daily maximum temperature, and daily minimum temperature separately. The results, not shown, indicated that simulations based on parameterizations using daily mean temperature and daily maximum temperature generally exhibited closer alignment in terms of accuracy, both outperforming the results obtained from parameterization using daily minimum temperature. In this study, we exclusively utilized daily mean temperature for modeling and forecasting. Given that warming amplifies differences between models, the influence of temperature asymmetry on model performance under climate change scenarios deserves investigation.

## 5 Conclusion

Overall, the average correlation coefficient between the predicted SOS by the 13 models and the observed values exceeded 0.72, with the highest reaching 0.80. All models performed better than the NULL model, and the M1 model performed the best for all of the tree species. Under the SSP126 scenario, the average SOS advanced initially and then gradually delayed, with a turning point around 2070. In the SSP585 scenario, the average SOS gradually advanced at a rate of approximately 0.14 days per year. Under the SSP126 scenario, the models exhibited relatively small and stable standard deviations. In the SSP585 scenario, from 2021 to 2100, the standard deviation of the simulated SOS by the 13 spring phenology models showed a significant increase, with an average annual increase of 0.04 days. On average, compared to one-phase models, two-phase models exhibited larger standard deviations after approximately 2050. Compared to models driven by both temperature and photoperiod, models driven solely by temperature showed larger standard deviations after 2060. Our results emphasize the differences in existing phenological models under warming scenarios and suggest investigating the release mechanisms of the endodormancy phase and incorporating new insights into future phenological models to better simulate changes in plant phenology.

## Data availability statement

The original contributions presented in the study are included in the article/**Supplementary Material**. Further inquiries can be directed to the corresponding author.

## Author contributions

YM: Data curation, Formal Analysis, Methodology, Software, Validation, Visualization, Writing – original draft, Writing – review & editing. XL: Writing – review & editing, Investigation, Methodology. YG: Writing – review & editing. YF: Writing – review & editing, Funding acquisition, Supervision, Conceptualization.
