# Supplementary material for: Warming increases the differences among spring phenology models under future climate change
Source: Front Plant Sci. 2023 Oct 23;14:1266801. doi: 10.3389/fpls.2023.1266801 (PMC10626552; doi:10.3389/fpls.2023.1266801)
Supplement: Supplementary file 1 [file DataSheet_1.pdf]

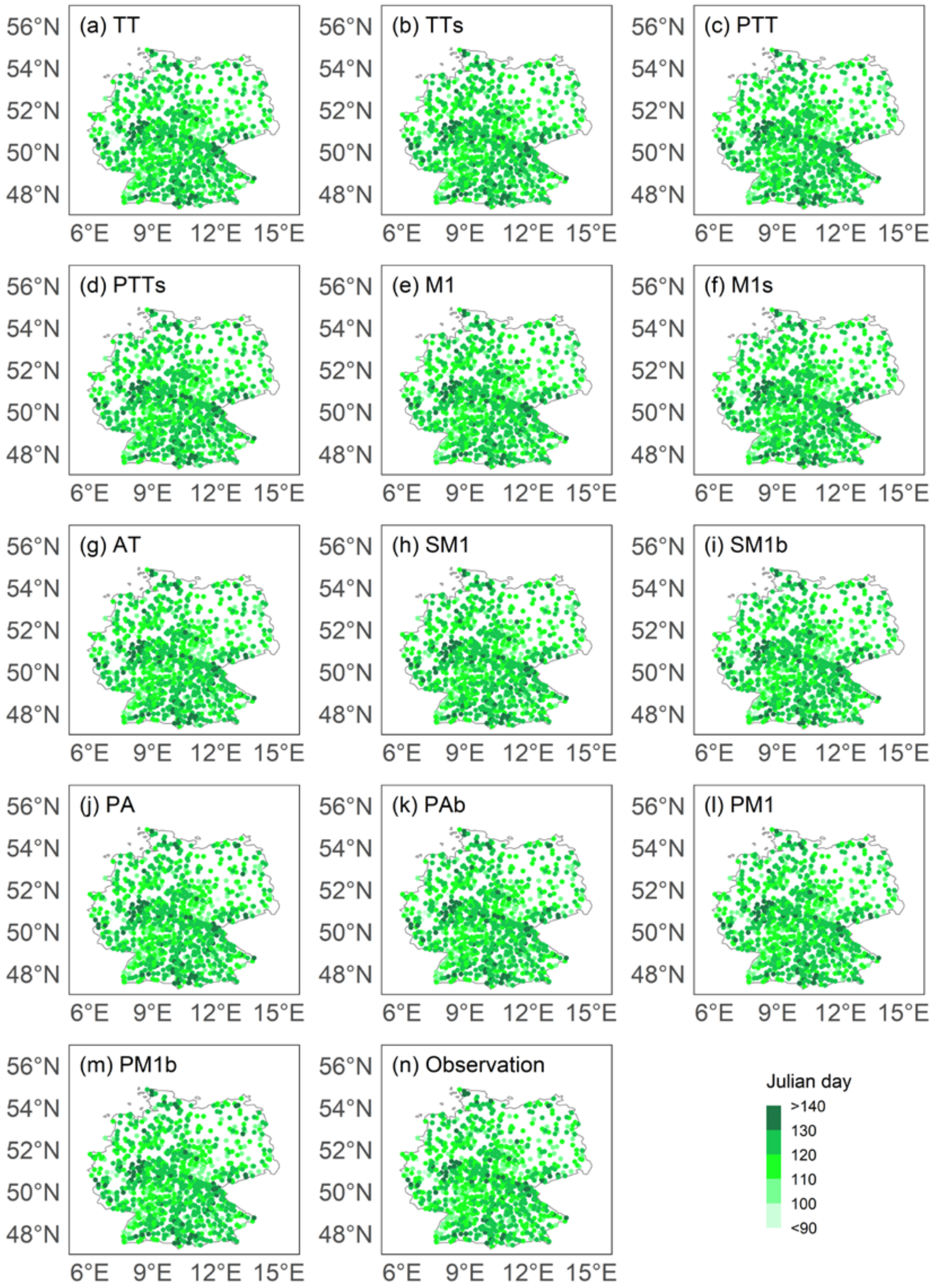

Supplementary Figure 1. The spatial patterns of the multiyear mean start of the growing season (SOS) predicted by 13 spring phenological models (a-m) and the PEP727 phenological observations (n).

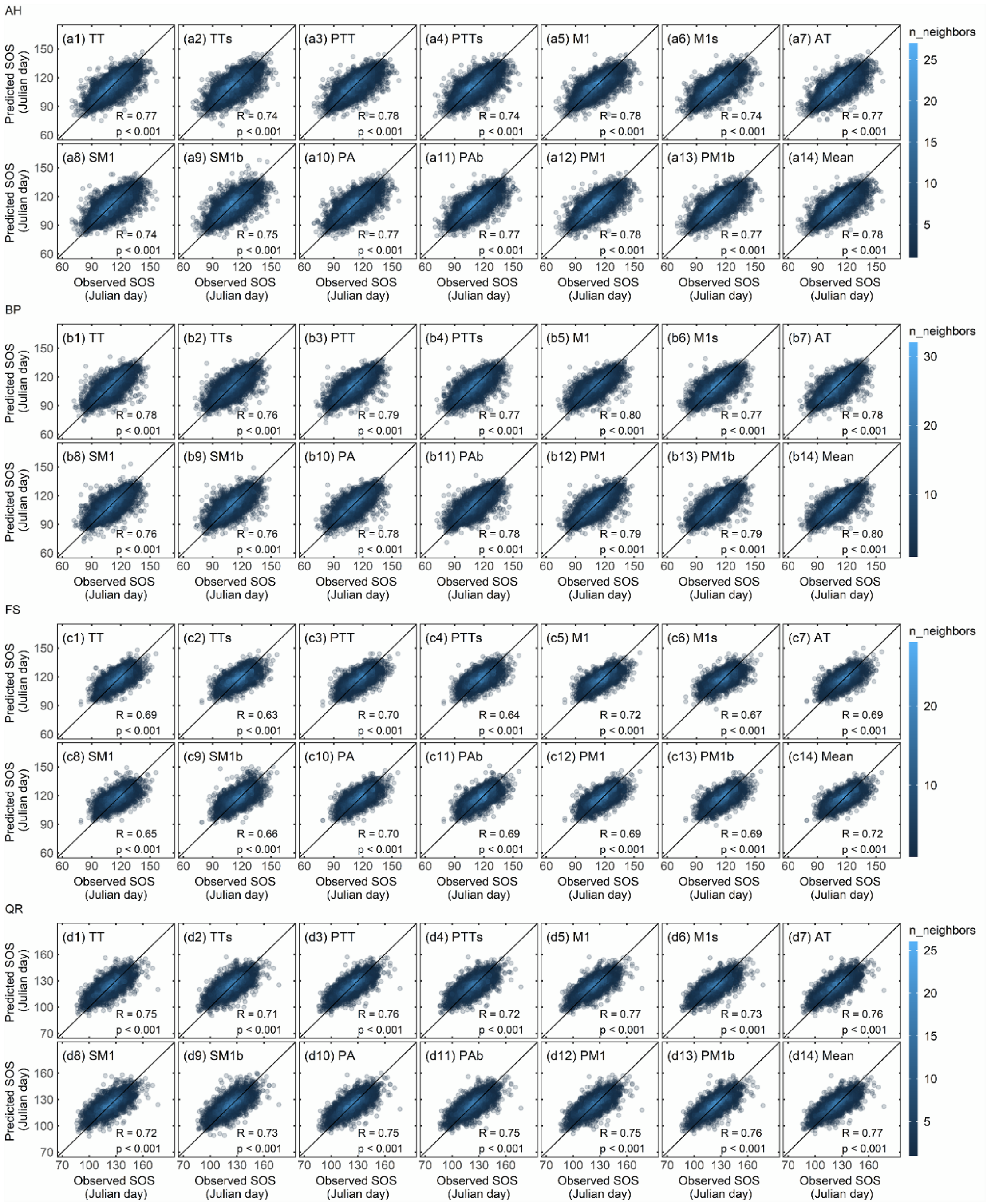

Supplementary Figure 2. Scatter plot of the model-predicted start of growing season (SOS) and PEP725 phenological observation. Figures a1-a14, b1-b14, c1-c14, and d1-d14 show the mean prediction results of 13 models and their mean of Aesculus hippocastanum (AH), Betula pendula (BP), Fagus sylvatica (FS), and Quercus robur (QR), respectively. The solid line in each figure is a 1:1 line. n_neighbors reflects the degree of aggregation of data points (each data point is connected to its n nearest neighbors).

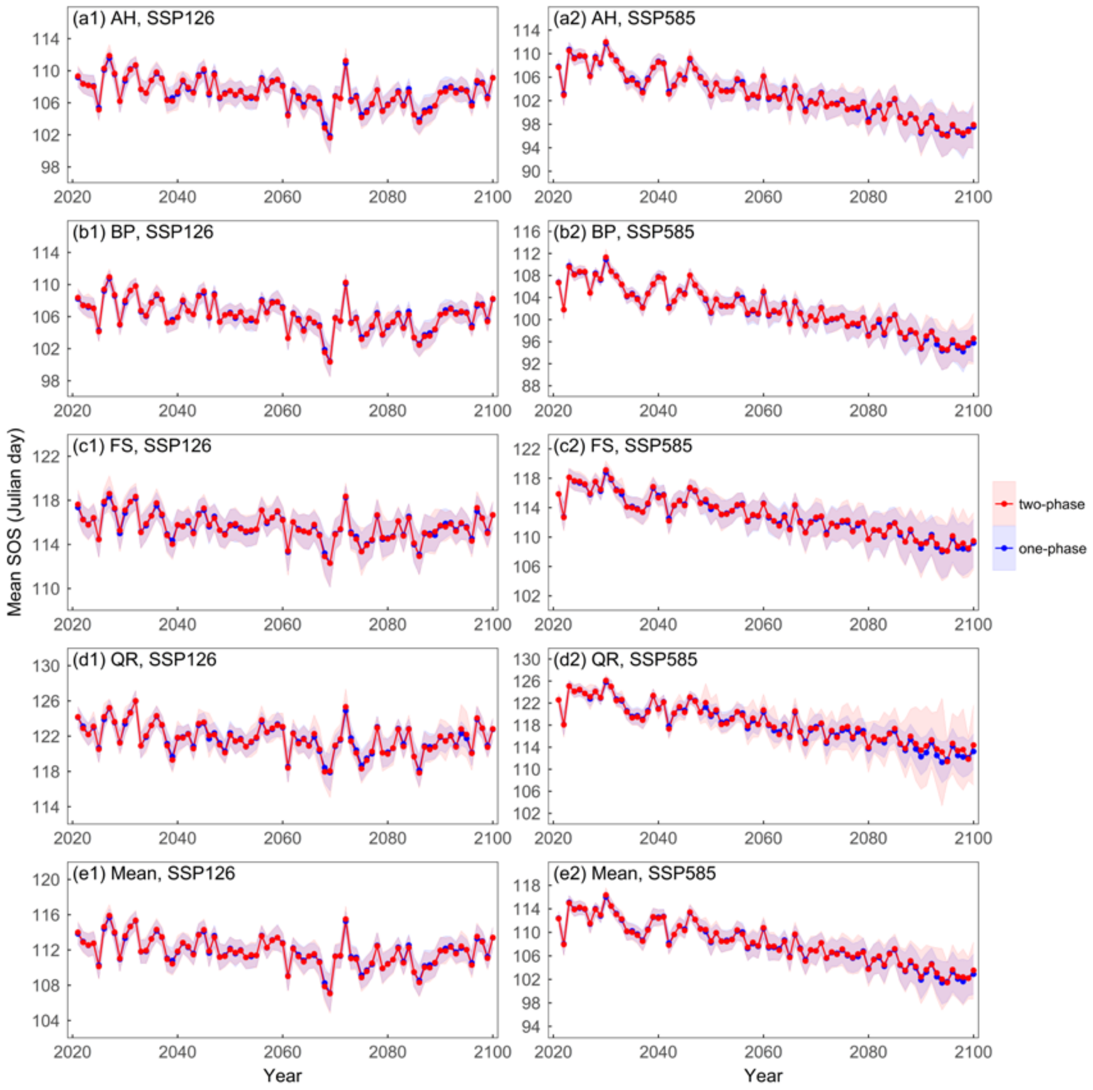

Supplementary Figure 3. The impact of dormancy release on the model-predicted start of growing season (SOS) for the four tree species: (a) Aesculus hippocastanum (AH), (b) Betula pendula (BP), (c) Fagus sylvatica (FS), (d) Quercus robur (QR), and (e) their mean. The shading represents the standard deviation of SOS predicted by either the one-phase models or the two-phase models.

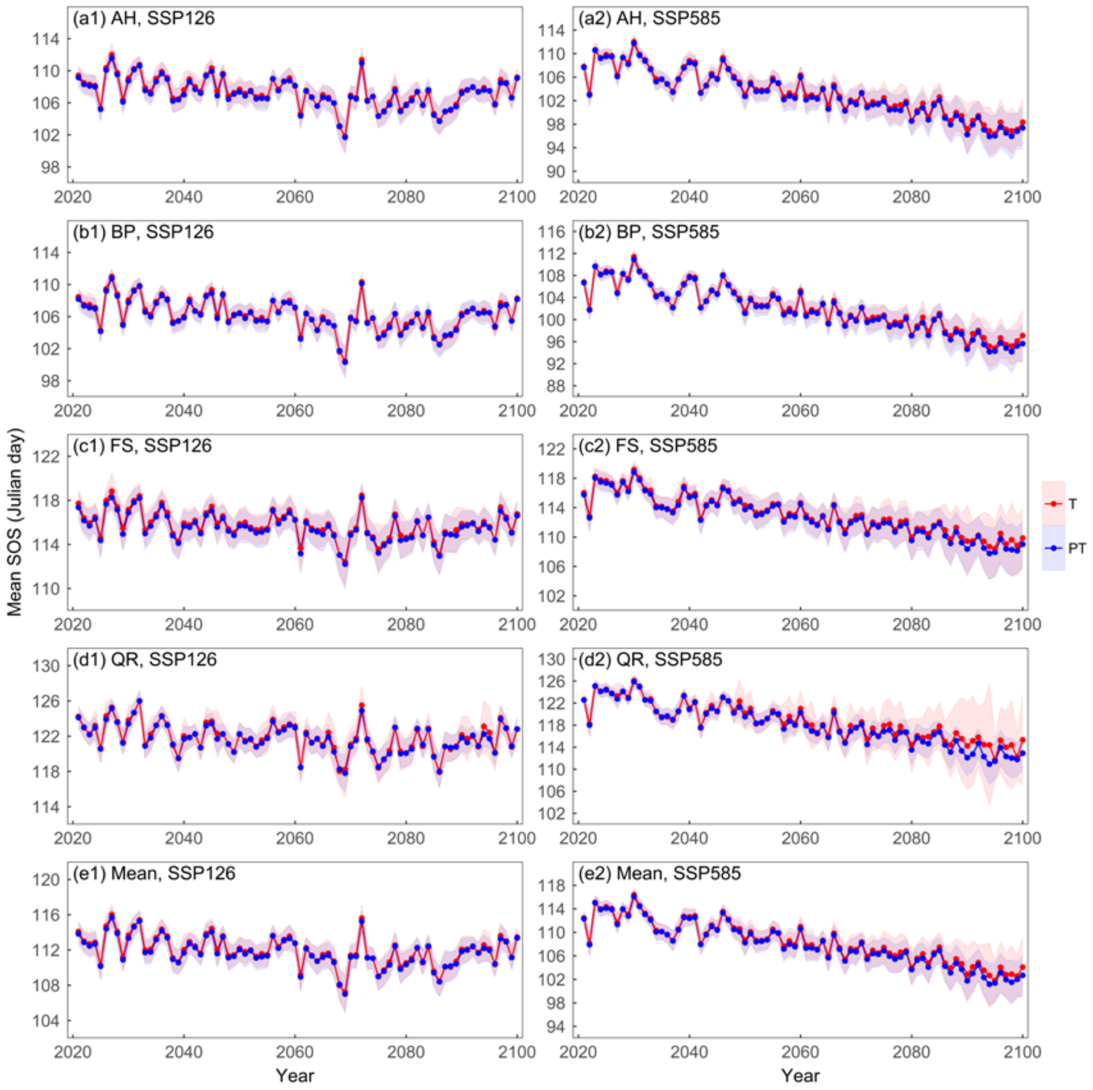

Supplementary Figure 4. The impact of driving factors on the model-predicted start of growing season (SOS) for the four tree species: (a) Aesculus hippocastanum (AH), (b) Betula pendula (BP), (c) Fagus sylvatica (FS), (d) Quercus robur (QR), and (e) their mean. The shading represents the standard deviation of the predicted SOS using either temperature (T)-driven models or models driven by both temperature and photoperiod (PT) simultaneously.

Supplementary Table 1. CMIP6 models used in this study.

CMIP6 models	Grid spacing	Institution
BCC-CSM2-MR	1.125° × 1.125°	Beijing Climate Center (China)
CanESM5	2.8125° × 2.8125°	Canadian Centre for Climate Modelling and Analysis (Canada)
FGOALS-g3	2° × 2.25°	Institute of Atmospheric Physics, Chinese Academy of Sciences (China)
GFDL-ESM4	1.25° × 1°	Geophysical Fluid Dynamics Laboratory, National Oceanic and Atmosphere Administration (USA)
INM-CM4-8	2° × 1.5°	Institute for Numerical Mathematics (Russia)
INM-CM5-0	2° × 1.5°	Institute for Numerical Mathematics (Russia)
IPSL-CM6A-LR	2.5° × 1.2587°	Institute Pierre Simon Laplace (France)
MIROC6	1.40625° × 1.40625°	Japan Agency for Marine-Earth Science and Technology (Japan)
MPI-ESM1-2-HR	0.9375° × 0.9375°	Max Planck Institute for Meteorology (Germany)
MPI-ESM1-2-LR	1.875° × 1.875°	Max Planck Institute for Meteorology (Germany)
MRI-ESM2-0	1.125° × 1.125°	Meteorological Research Institute, Japan Meteorological Agency (Japan)
NorESM2-LM	2.5° × 1.875°	Norwegian Climate Center (Norway)
NorESM2-MM	1.25° × 0.9375°	Norwegian Climate Center (Norway)

Supplementary Table 2. Temperature response functions and structures of chilling/forcing-based spring phenology models used in this study. The models are driven by daily mean temperature (T_i) and photoperiod (L_i) after a starting date t_0. The endodormancy release uses a triangular temperature response (r_t) or a bell-shaped temperature response (r_b), and the ecodormancy release uses a growing-degree-day temperature response (r_g) or a sigmoid temperature response (r_s). The M1 model introduces the effect of photoperiod based on r_g. See Supplementary Table 3 for a description of the symbols in the functions. For the NULL model, *OBS_i* is the *i*-th observation, *n* is the number of observations.

temperature response function	Chilling temperature response		
	Triangular temperature response	Bell-shaped temperature response	
	$r_t(T_i) = \begin{cases} \frac{T_i - T_n}{T_{opt} - T_n} & T_n \le T_i < T_{opt} \\ 1 - \frac{T_i - T_{opt}}{T_x - T_{opt}} & T_{opt} \le T < T_x \\ 0 & T_i < T_n \mid T_i > T_x \end{cases}$	$r_b(T_i) = \frac{1}{1 + e^{(a*(T_i - c)^2 + b*(T_i - c))}}$	
	Forcing temperature response		
	Growing-degree-day temperature response	Sigmoid temperature response	
	$r_g(T_i) = \begin{cases} 0 & T_i \le T_{base} \\ T_i - T_{base} & T_i > T_{base} \end{cases}$	$r_s(T_i) = \frac{1}{1 + e^{(-b*(T_i - c))}}$	
	State of chilling	State of forcing	Criteria for budburst
	$S_{chl} = \sum_{i=t_0}^{n} R_{chl}$	$S_{frc} = \sum_{i=t_0}^{n} R_{frc}$	$S_{frc} \ge F_{crit}$
Ecodormancy release	Thermal Time model (TT)	Sigmoid Thermal Time model (TTs)	
	$R_{frc} = r_g$	$R_{frc} = r_s$	
	Photo Thermal Time model (PTT)	Sigmoid Photo Thermal Time model (PTTs)	

	$R_{frc} = \frac{L_i}{24} r_g$	$R_{frc} = \frac{L_i}{24} r_s$
	M1 model (M1)	Sigmoid M1 model (M1s)
	$R_{frc} = (\frac{L_i}{10})^k r_g$	$R_{frc} = (\frac{L_i}{10})^k r_s$
Endo-and ecodormancy releases	Sequential models (SM1)	Sequential models (SM1b)
	$R_{chl} = r_t$	$R_{chl} = r_b$
	$R_{frc} = (\frac{L_i}{24})^k r_g$	$R_{frc} = (\frac{L_i}{24})^k r_g$
	$k = \begin{cases} 0 & S_{chl} < C_{req} \\ 1 & S_{chl} \geq C_{req} \end{cases}$	$k = \begin{cases} 0 & S_{chl} < C_{req} \\ 1 & S_{chl} \geq C_{req} \end{cases}$
	Parallel models (PA)	Parallel models (PAb)
	$R_{chl} = r_t$	$R_{chl} = r_b$
	$R_{frc} = k r_g$	$R_{frc} = k r_g$
	$k = \begin{cases} C_{ini} + S_{chl} \frac{1 - C_{ini}}{C_{req}} & S_{chl} < C_{req} \\ 1 & S_{chl} \geq C_{req} \end{cases}$	$k = \begin{cases} C_{ini} + S_{chl} \frac{1 - C_{ini}}{C_{req}} & S_{chl} < C_{req} \\ 1 & S_{chl} \geq C_{req} \end{cases}$
	Parallel models (PM1)	Parallel models (PM1b)
	$R_{chl} = r_t$	$R_{chl} = r_b$
	$R_{frc} = (\frac{L_i}{10})^k r_g$	$R_{frc} = (\frac{L_i}{10})^k r_g$
	$k = \begin{cases} C_{ini} + S_{chl} \frac{1 - C_{ini}}{C_{req}} & S_{chl} < C_{req} \\ 1 & S_{chl} \geq C_{req} \end{cases}$	$k = \begin{cases} C_{ini} + S_{chl} \frac{1 - C_{ini}}{C_{req}} & S_{chl} < C_{req} \\ 1 & S_{chl} \geq C_{req} \end{cases}$

Alternating model (AT)	NULL model
$R_{chl} = \begin{cases} 0 & T_i < T_{base} \\ 1 & T_i \geq T_{base} \end{cases}$<br>$R_{frc} = r_g$<br>$F_{crit} = a + b * e^{c*S_{chl}}$	$\frac{\sum_{i=1}^{n} OBS_i}{n}$

Supplementary Table 3. Description of the symbols in the functions in Supplementary Table 2.

Symbol	Description of the Symbol	Units
Variables		
r_t	Triangular temperature response for chilling during endodormancy release	-
r_b	Bell-shaped temperature response for chilling during endodormancy release	-
r_g	Growing-degree-day temperature response for forcing during ecodormancy release	-
r_s	Sigmoid temperature response for forcing during ecodormancy release	-
R_{chl}	Rate of chilling	-
R_{frc}	Rate of forcing	-
S_{chl}	State of chilling, integral of rate of chilling	-
S_{frc}	State of forcing, integral of rate of forcing	-
k	Competence function: bud's potential to respond to forcing temperature	-
T_i	Daily temperature	℃
L_i	Daily photoperiod	h
t_0	Starting date	day
Parameters		
T_n	Minimum temperature for rate of chilling	℃
T_{opt}	Optimal temperature for rate of chilling	℃

T_x	Maximum temperature for rate of chilling	℃
T_{base}	Base temperature	℃
C_{ini}	Minimum potential of unchilled bud to respond to forcing temperature	-
C_{req}	Requirement value of state of chilling for the transition from endodormancy to ecodormancy	-
F_{crit}	Critical values of state of forcing for the transition from ecodormancy to budburst	-
a, b, c	Constants	-
